# Small Bowel-Vesical Fistula with Chronic Kidney Disease Relating to Administration of Sodium Polystyrene Sulfonate: A Case Report

**DOI:** 10.70352/scrj.cr.26-0641

**Published:** 2026-10-24

**Authors:** Hirokazu Kawahara, Toshifumi Matsumoto, Hiroki Orimoto, Yuichi Nakazono, Teijiro Hirashita, Tomonori Akagi, Masafumi Inomata

**Affiliations:** 1 Department of Surgery, NHO Beppu Medical Center, Beppu, Oita, Japan; 2 Department of Gastroenterological and Pediatric Surgery, Oita University Faculty of Medicine, Yufu, Oita, Japan; 3 Department of Pathology, NHO Beppu Medical Center, Beppu, Oita, Japan

**Keywords:** sodium polystyrene sulfonate, small bowel-vesical fistula, chronic kidney disease

## Abstract

**INTRODUCTION:**

Sodium polystyrene sulfonate (SPS) is a cation-exchange resin indicated for the treatment of hyperkalemia in patients with chronic kidney disease (CKD); however, it is associated with severe gastrointestinal complications such as intestinal perforation, ulceration, and necrosis. Although SPS-related gastrointestinal injury has been well documented, small bowel-vesical fistula has not previously been reported. Here, we present a case of small bowel-vesical fistula associated with SPS therapy.

**CASE PRESENTATION:**

A 69-year-old man with CKD who was receiving SPS therapy presented to a local clinic with the passage of food particles in his urine. The patient was subsequently referred to our institution for further evaluation and management. CT revealed a fistula between the small intestine and the urinary bladder, prompting surgical intervention. Partial resection of the small bowel and partial cystectomy were performed to excise the fistulous tract. Postoperatively, the patient developed an anastomotic leak in the small bowel that required ileostomy. His condition subsequently improved, and he was discharged from the hospital. Histopathological examination of the tissue surrounding the fistula demonstrated an inflammatory infiltrate predominantly composed of plasma cells, along with numerous basophilic polygonal crystals consistent with SPS. These pathological findings supported an association between SPS deposition and intestinal injury as a possible underlying mechanism of small bowel-vesical fistula formation. To our knowledge, this is the first reported case of small bowel-vesical fistula associated with SPS therapy.

**CONCLUSIONS:**

Patients with CKD are susceptible to SPS-associated gastrointestinal injury because of underlying intestinal ischemia. Clinicians should be aware of small bowel-vesical fistula as a rare but serious complication associated with SPS therapy, and proactive bowel management may help prevent its development.

## Abbreviations

AKI
: acute kidney injury
CBD
: common bile duct
CKD
: chronic kidney disease
CPS
: calcium polystyrene sulfonate
SPS
: sodium polystyrene sulfonate

## INTRODUCTION

SPS is a cation-exchange resin used to treat hyperkalemia by adsorbing potassium ions in the intestinal tract and facilitating their fecal excretion. Although SPS is widely used to manage hyperkalemia in patients with AKI and CKD, severe gastrointestinal adverse events, including perforation, ulceration, and necrosis, have been reported with both SPS and CPS.^1,2)^ However, small bowel-vesical fistula associated with SPS therapy has not been previously reported. Herein, we report a rare case of small bowel-vesical fistula that developed in a patient with CKD receiving oral SPS therapy and review the relevant literature.

## CASE PRESENTATION

A 69-year-old man with CKD on maintenance hemodialysis presented to a local clinic with the passage of food particles in his urine, 3 months after conservative treatment for a urinary tract infection. A small bowel-vesical fistula was suspected, prompting referral to our institution for further evaluation and management. His medical history was significant for ileocecal resection for colonic tuberculosis at 22 years of age, inactive pulmonary tuberculosis, hypertension, and constipation. Because of CKD with hyperkalemia, the patient had been receiving oral SPS. The SPS formulation was Kayexalate syrup 76% (3 packets 3 times daily; SPS equivalent, 7.5 g/day), which did not contain sorbitol. Because this medication had been prescribed at another hospital, the exact duration of administration was unknown. On admission, physical examination revealed a soft, nondistended abdomen with a surgical scar. Laboratory findings demonstrated no marked inflammatory response (C-reactive protein, 0.52 mg/dL; white blood cell count, 4950/μL), with a blood urea nitrogen level of 20.0 mg/dL, a serum creatinine level of 3.11 mg/dL, and a serum potassium level of 3.9 mmol/L. Urinalysis was positive for occult blood and leukocytes. CT performed 3 months earlier demonstrated multiple hyperdense fecal masses within dilated pelvic small bowel loops (**Fig. 1**). Follow-up CT at presentation showed persistent hyperdense fecal material together with intravesical gas (**Fig. 2A**) and inflammatory soft tissue bridging the small bowel and bladder, consistent with a small bowel-vesical fistula (**Fig. 2B**–**2D**). Incidentally, gallstones and a CBD stone were identified. The treatment plan commenced with endoscopic removal of the CBD stone, followed by definitive surgery. Upon laparotomy, extensive adhesions between the omentum and small bowel resulting from the previous surgery were divided to obtain adequate surgical exposure. The distal ileum was firmly adherent to the bladder, and a fistulous tract was identified. The fistula and the adjacent indurated bladder wall were resected en bloc, and the defect was repaired in 2 layers using continuous sutures. Partial resection of the small bowel was performed with a hand-sewn end-to-end anastomosis, and the procedure was completed with a cholecystectomy following dissection of the pericholecystic adhesions. On POD 5, a follow-up CT revealed free air in the abdominal cavity. Emergency laparotomy was performed, which revealed a small bowel anastomotic leak. The anastomotic site was resected, and an ileostomy was fashioned using the proximal ileal stump. The patient recovered with rehabilitation and was discharged in stable condition. Histopathological examination of the resected specimen, which consisted of a 25-cm U-shaped segment of the small bowel adherent to a portion of the bladder wall (**Fig. 3**), revealed a deep ulcer in the small bowel wall penetrating the muscularis propria and forming a fistula that reached the bladder mucosa. The area surrounding the fistula exhibited marked inflammatory infiltration, predominantly composed of plasma cells (**Fig. 4A**). Numerous characteristic basophilic polygonal crystals measuring approximately 10–100 μm in diameter were identified within the inflammatory infiltrate, consistent with SPS crystals (**Fig. 4B**). These pathological findings supported the diagnosis of SPS-associated intestinal injury, with associated chronic ulceration and small bowel-vesical fistula formation.

**Fig. 1:**
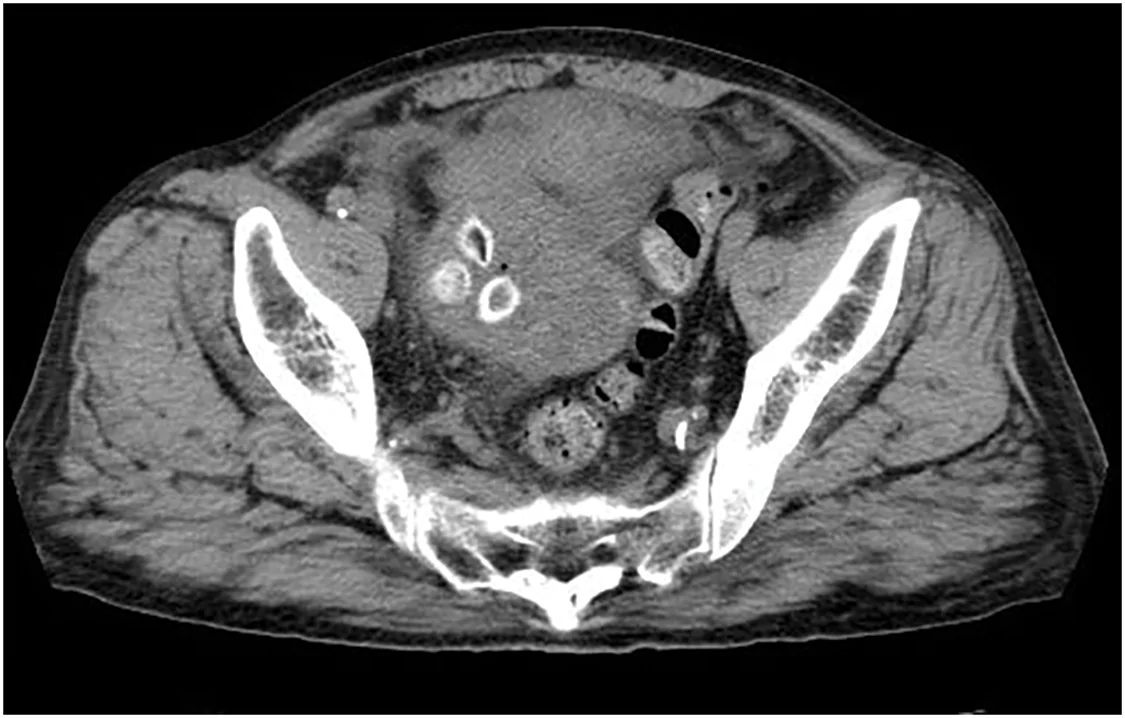
CT findings obtained 3 months before presentation. Hyperdense fecal masses are observed within the small bowel.

**Fig. 2:**
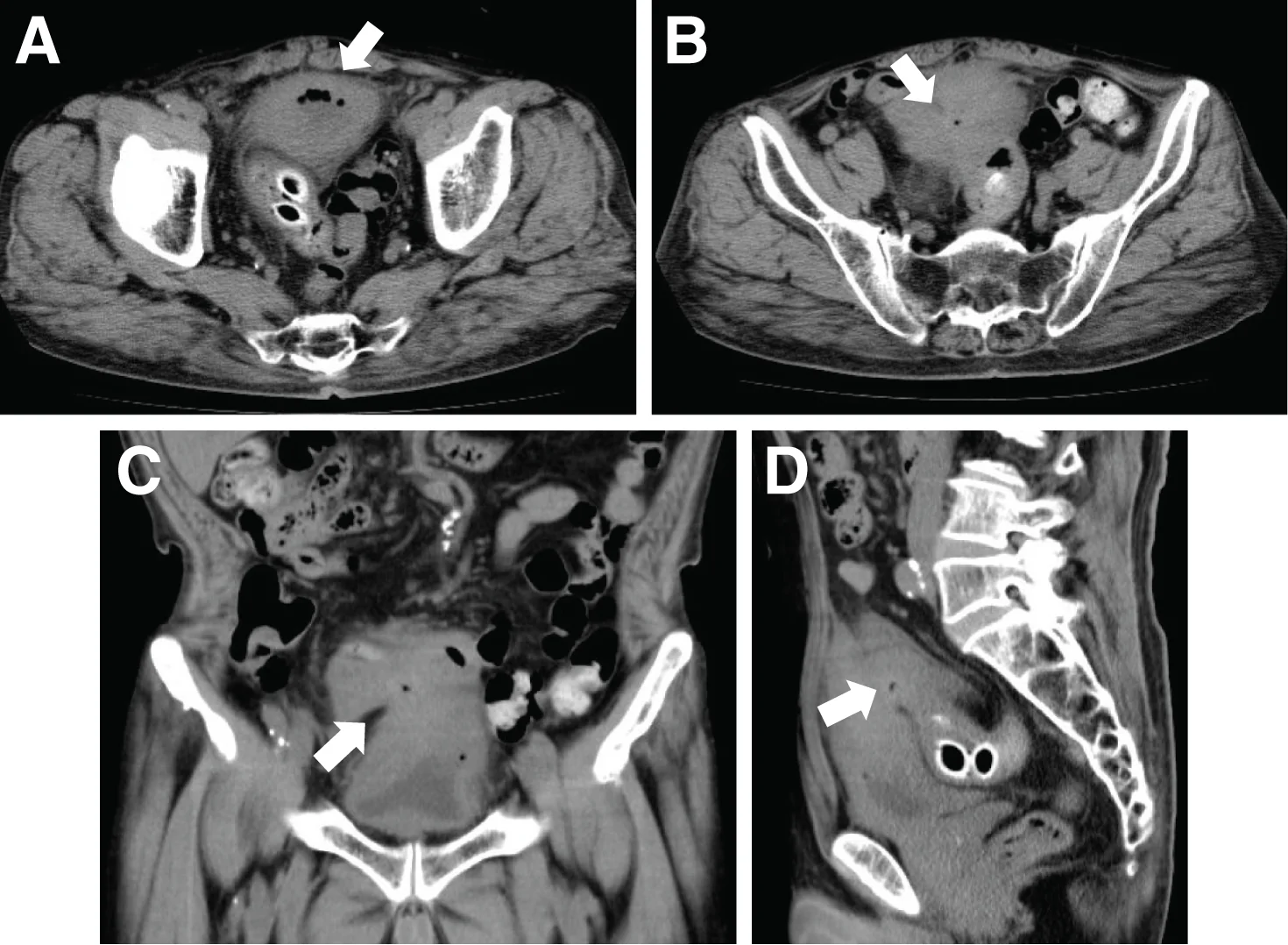
CT findings at the time of referral. (**A**) Persistent hyperdense fecal masses are observed within the small bowel, with gas present within the urinary bladder (arrow). A small bowel-vesical fistula (arrows) is shown in (**B**) axial, (**C**) coronal, and (**D**) sagittal views.

**Fig. 3:**
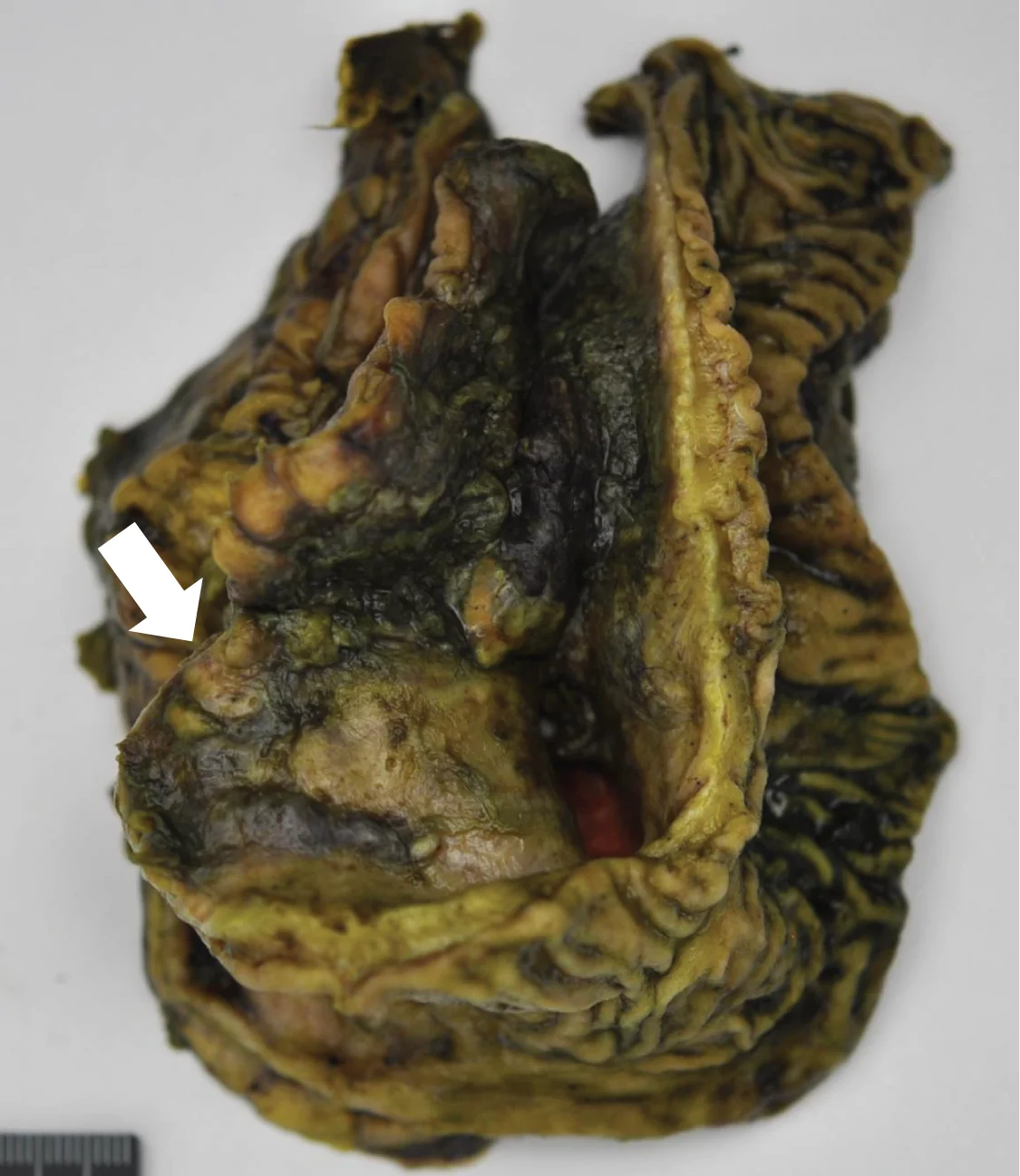
Resected specimen. Gross appearance showing an approximately 25-cm, U-shaped, adherent segment of the small bowel and a portion of the bladder wall (arrow).

**Fig. 4:**
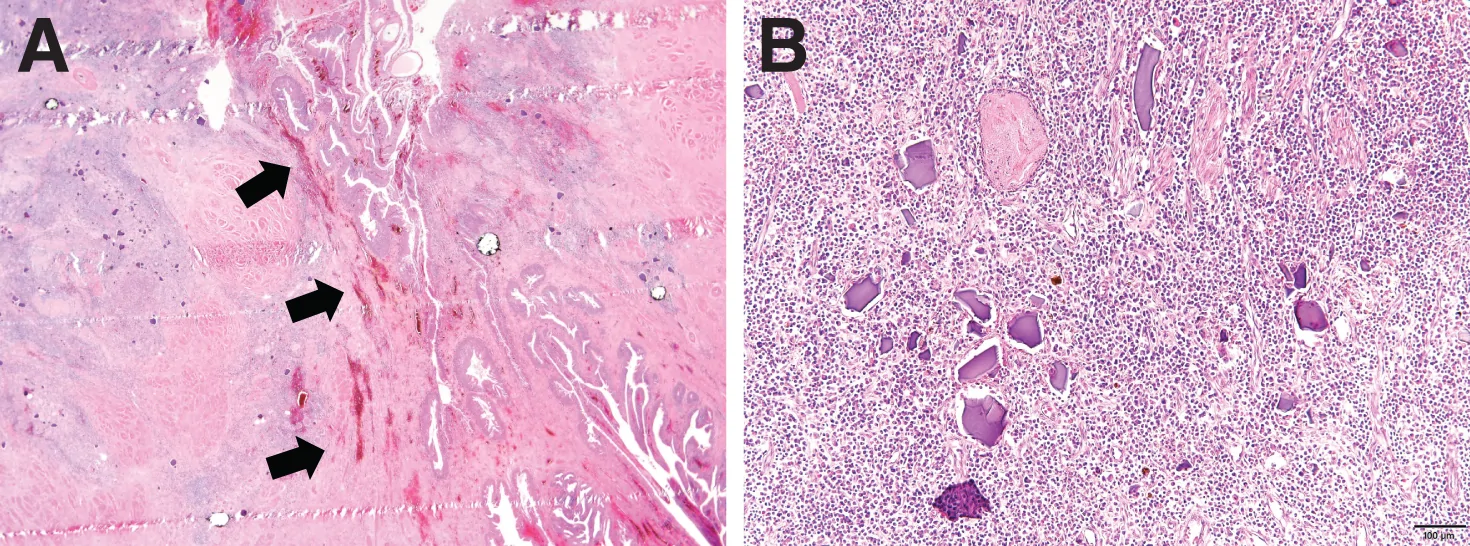
Histopathological findings (hematoxylin and eosin stain). (**A**) Low-power view: The fistulous tract extends to the bladder mucosa (arrows). (**B**) High-power view: Deposition of numerous basophilic polygonal crystals, measuring approximately 10–100 μm, is observed, surrounded by abundant inflammatory cells.

## DISCUSSION

SPS and CPS are cation-exchange resins widely used for the treatment of hyperkalemia in patients with CKD; however, they have been associated with serious gastrointestinal complications, including ulceration, necrosis, perforation, and bleeding.^1,2)^ Because these nonabsorbable resins form hard fecal masses within the intestinal lumen, prolonged retention may increase intraluminal pressure and cause mechanical injury to the bowel wall.^3)^ The left colon, particularly the sigmoid colon, is the most commonly affected site, whereas small bowel involvement is extremely rare.^4)^ Okayama et al. proposed a “2-hit theory” to explain this rare complication.^5)^ In patients with CKD, chronic mesenteric hypoperfusion caused by activation of the renin–angiotensin system and coexisting atherosclerotic disease results in initial ischemic mucosal injury (the first hit). SPS crystals subsequently penetrate and become deposited within the damaged mucosa, where persistent crystal-induced inflammation promotes mucosal shedding and transmural necrosis (the second hit). The preoperative CT findings in our patient, showing persistent hyperdense fecal masses, are consistent with this proposed mechanism, suggesting prolonged retention of SPS-containing feces and chronic injury with crystal deposition. Histopathological examination revealed SPS crystal deposition within the fistulous tract involving both the small bowel and bladder walls, accompanied by marked inflammatory cell infiltration, which is consistent with and supports this proposed mechanism in the present case.

We searched PubMed and Ichushi-Web (Japan Medical Abstracts Society) for case reports of small bowel injury caused by oral SPS or CPS published between January 2000 and December 2025, using the keywords “polystyrene sulfonate” and “intestine.” Reports of cases involving small bowel injury were scarce, with only 12 cases identified.^5–16)^ Previously reported cases of SPS-associated small bowel injury have predominantly presented with intestinal stenosis or ulceration, and none has described fistula formation into the urinary bladder. A vesical fistula associated with polystyrene sulfonate administration has been reported previously; however, that case involved the sigmoid colon (a sigmoidovesical fistula caused by CPS with sorbitol), and the patient underwent sigmoidectomy together with partial resection of the ileum and bladder.^17)^ In contrast, the fistula in our patient arose exclusively from the small bowel, without colonic involvement. To the best of our knowledge, this is the first reported case of a small bowel-vesical fistula associated with SPS therapy, rather than the first report of an enterovesical fistula associated with SPS therapy in general.

We speculate that several patient-specific factors contributed to this unusual small bowel–vesical presentation, rather than the more commonly reported colonic stenosis or gastrointestinal bleeding. Because of the patient’s previous ileocecal resection, the small bowel, normally freely mobile within the abdominal cavity, had become fixed by postoperative adhesions adjacent to the bladder; chronic SPS-associated ulceration at this fixed site may have progressed to fistula formation into the bladder rather than manifesting as intestinal stenosis or gastrointestinal bleeding.

Histopathological examination of the small bowel segment resected during the present surgery did not reveal any findings suggestive of active or recurrent tuberculosis, such as caseating granulomas or acid-fast bacilli. In addition, given that the patient’s pulmonary tuberculosis had become inactive and no clinical signs of active intestinal tuberculosis were present at the current admission, active or recurrent intestinal tuberculosis was considered an unlikely contributor to the present pathology. Under normal physiological conditions, the ileocecal valve prevents reflux between the colon and the small bowel. Loss of this barrier following ileocecal resection may permit retrograde movement of colonic contents into the small bowel. We speculate that, in combination with severe constipation, this anatomical alteration promoted prolonged stagnation of SPS-containing fecal material within the distal small bowel, facilitating persistent mucosal contact, crystal deposition, and subsequent intestinal injury.

In addition, the location of the prior ileocecal anastomosis was not specifically identified or documented at the time of the current surgery. If the prior ileocecal anastomosis and the site of the current anastomosis were located in close proximity, the present small bowel resection may have been performed in the intervening segment, and the possibility of relatively compromised mesenteric blood supply in this segment cannot be excluded. The exact distance between the prior ileocecal anastomosis and the site of the present fistula and anastomosis was not measured intraoperatively. We did not perform an intraoperative assessment of mesenteric blood flow (e.g., indocyanine green fluorescence angiography) at either anastomotic site, and no direct evidence of ischemia was documented at the time of surgery; this remains speculative and is discussed further below in relation to the postoperative anastomotic leak.

Furthermore, the patient developed a postoperative anastomotic leak requiring reoperation. The patient was undergoing hemodialysis for CKD and receiving antihypertensive therapy, both of which are associated with chronic mesenteric hypoperfusion, as discussed above in relation to the “2-hit theory”.^5)^ We therefore speculate that subclinical microvascular ischemia, rather than SPS crystal deposition itself, was the primary factor predisposing to impaired anastomotic healing; SPS deposition, when present, is more likely to be a secondary consequence of such ischemic mucosal injury than an independent cause of leakage. However, no intraoperative assessment of mesenteric blood flow was performed at either anastomotic site, and the resected specimen from the reoperation was not histopathologically examined for SPS crystal deposition or ischemic change. In the absence of direct histopathological or intraoperative evidence at the anastomotic margins, this causal relationship remains speculative.

CKD itself is a recognized risk factor for anastomotic leakage after gastrointestinal surgery in general, independent of SPS use.^18)^ By contrast, we are not aware of any published reports specifically identifying SPS or CPS use as a risk factor for anastomotic leakage following intestinal resection. Therefore, careful intraoperative assessment of bowel viability, including consideration of blood flow evaluation techniques such as indocyanine green fluorescence imaging, and cautious selection of the anastomotic site are warranted in patients receiving SPS who also have CKD-related risk factors for impaired anastomotic healing.

Careful bowel management is essential to prevent SPS-associated gastrointestinal complications. Patients with CKD receiving SPS are particularly susceptible to constipation because of fluid restriction, intraluminal dehydration associated with hemodialysis, and reduced dietary fiber intake resulting from potassium-restricted diets.^19)^ In the present case, magnesium oxide was used as a laxative; however, its use should be carefully monitored because of the risk of hypermagnesemia in patients with renal impairment. When conventional laxatives are insufficient or unsuitable, newer agents, including intestinal secretagogues and ileal bile acid transporter inhibitors, may represent useful alternatives for maintaining adequate bowel function and reducing prolonged intestinal retention of SPS.

## CONCLUSIONS

Herein, we report a case of a small bowel-vesical fistula associated with SPS therapy. Patients with CKD are highly susceptible to gastrointestinal ischemia, which may predispose them to SPS deposition and associated gastrointestinal injury. Given that constipation can exacerbate this proposed pathogenic process, proactive bowel management is essential.

## DECLARATIONS

### Availability of data and materials

The data that support the findings of this study are available from the corresponding author upon reasonable request.
